# A qualitative exploration of the salience of MTV-Shuga, an edutainment programme, and adolescents’ engagement with sexual and reproductive health information in rural KwaZulu-Natal, South Africa

**DOI:** 10.1080/26410397.2022.2083809

**Published:** 2022-08-05

**Authors:** Nambusi Kyegombe, Thembelihle Zuma, Siphesihle Hlongwane, Mxolisi Nhlenyama, Natsayi Chimbindi, Isolde Birdthistle, Sian Floyd, Janet Seeley, Maryam Shahmanesh

**Affiliations:** a Associate Professor, Department of Global Health and Development, London School of Hygiene and Tropical Medicine, London, UK. *Correspondence:* nambusi.kyegombe@lshtm.ac.uk; b Senior Research Associate, Africa Health Research Institute, KwaZulu-Natal, South Africa; c Research Professional, Africa Health Research Institute, KwaZulu-Natal, South Africa; d Research Assistant, Africa Health Research Institute, KwaZulu-Natal, South Africa; e Junior Faculty, Africa Health Research Institute, KwaZulu-Natal, South Africa; f Associate Professor, Department of Population Health, London School of Hygiene and Tropical Medicine, London, UK; g Professor, Department of Global Health and Development, London School of Hygiene and Tropical Medicine, London, UK; h Senior Faculty, Africa Health Research Institute, KwaZulu-Natal, South Africa; i Professor, Institute of Global Health, University College London; j Senior Faculty and Study PI, Africa Health Research Institute, KwaZulu-Natal, South Africa

**Keywords:** MTV Shuga, edutainment, sexual and reproductive health, adolescent girls and young women, adolescent boys and young men, South Africa

## Abstract

Young people, particularly adolescent girls and young women, represent a growing proportion of those living with HIV. Edutainment programmes have been widely used throughout the world to “educate” and “entertain” audiences and tackle serious social issues in bold and engaging ways. This paper examines the extent to which an edutainment programme, MTV-Shuga, was reported to influence young people’s engagement with sexual and reproductive health (SRH) information in rural KwaZulu-Natal, South Africa. In 2019 we conducted eight community-based screenings of MTV-Shuga episodes followed by 25 individual in-depth interviews and 13 focus group discussions with young people aged between 15 and 30. Interviews were audio recorded and transcribed verbatim. Data analysis was thematic and complemented by constant comparison and deviant case analysis techniques. In this rural and poor setting with a high burden of HIV, young people exhibited high levels of awareness of SRH and HIV but had constrained access to services, and limited ability to engage with parents or guardians on SRH matters. MTV Shuga provided an entertaining guide of ways to navigate the risks that they faced in a way that resonated with them. The findings highlight the importance of enabling young people in rural areas to watch MTV Shuga with peers in a safe space in which discussion of the content is facilitated. There is also value in encouraging parents to watch MTV Shuga as a means of enabling discussions between children and adults in their lives about SRH matters.

## Résumé

Les jeunes, en particulier les adolescentes et les jeunes femmes, représentent une proportion croissante des personnes vivant avec le VIH. Les programmes ludo-éducatifs ont été largement utilisés dans le monde pour « éduquer » et « divertir » des publics et aborder de graves questions sociales avec audace et de manière agréable. Cet article examine dans quelle mesure un programme ludo-éducatif, la série télévisée MTV-Shuga, a influencé la participation des jeunes aux informations sur la santé sexuelle et reproductive dans la région du KwaZulu-Natal, en Afrique du Sud. En 2019, nous avons réalisé huit projections communautaires d’épisodes de MTV-Shuga suivies de 25 entretiens individuels approfondis et 13 discussions par groupe thématique avec des jeunes âgés de 15 à 30 ans. Les entretiens ont fait l’objet d’un enregistrement audio et ont été retranscrits textuellement. Le traitement des données était thématique et complété par des techniques de comparaison constante et d’analyse des cas déviants. Dans ce milieu rural et pauvre à forte charge de VIH, les jeunes affichaient des niveaux élevés de connaissance de la santé sexuelle et reproductive et du VIH, mais disposaient d’un accès limité aux services, et ils n’avaient guère de possibilités de dialoguer avec leurs parents ou leurs tuteurs sur les questions de santé sexuelle et reproductive. La série MTV Shuga a fourni un guide amusant sur les façons de gérer les risques qu’ils couraient d’une manière leur convenant. Les conclusions ont montré combien il est important de permettre aux jeunes des zones rurales de regarder MTV Shuga avec leurs pairs dans un espace sûr dans lequel la discussion du contenu est facilitée. Il est également utile d’encourager les parents à regarder MTV Shuga comme moyen de permettre les discussions entre enfants et adultes dans leur vie sur les questions de santé sexuelle et reproductive.

## Resumen

Las personas jóvenes, en particular las adolescentes y las mujeres jóvenes, representan un creciente porcentaje de aquéllas que viven con VIH. Los programas de educación y entretenimiento se han utilizado de manera extendida por todo el mundo para “educar” y “entretener” a públicos y encarar graves problemas sociales en formas audaces y atractivas. Este artículo examina en qué medida el programa de educación y entretenimiento, MTV-Shuga, se reportó para influir en la interacción de jóvenes con información sobre salud sexual y reproductiva en zonas rurales de KwaZulu-Natal, Sudáfrica. En 2019 realizamos ocho proyecciones comunitarias de episodios de MTV-Shuga seguidas de 25 entrevistas a profundidad individuales y 13 discusiones en grupos focales con jóvenes de 15 a 30 años. Las entrevistas se grabaron en audio y se transcribieron textualmente. El análisis de datos fue temático y suplementado por las técnicas de comparación constante y análisis de casos desviados. En este entorno rural y pobre con una carga elevada de VIH, las personas jóvenes exhibieron altos niveles de conocimiento de salud sexual y reproductiva y VIH, pero tenían acceso restringido a los servicios y posibilidad limitada de interactuar con sus padres o tutor/a en cuestiones de salud sexual y reproductiva. MTV Shuga ofreció una guía entretenida de formas de navegar los riesgos que enfrentaban de una manera que resonara con ellos. Los hallazgos destacan la importancia de permitir que las personas jóvenes en zonas rurales vean MTV Shuga con sus pares en un espacio seguro, donde se faciliten conversaciones sobre el contenido. Además, hay valor en alentar a los padres a ver MTV Shuga como medio de facilitar las conversaciones entre niños y adultos en su vida sobre asuntos de salud sexual y reproductiva.

## Background

Worldwide, adolescents and young people represent a growing proportion of those living with HIV.^1^ Adolescent girls and young women aged between 15 and 24 years and living in sub-Saharan Africa are at particular risk. Owing to multiple biological, social, and structural factors that increase their vulnerability and constrain their ability to avoid infection,^2^ adolescent girls and young women typically have HIV infection rates that are twice as high as their male counterparts.^3–5^ Indeed, 31% of all new infections in sub-Saharan Africa are estimated to be among this group.^3,4,6^ This picture is also reflected in South Africa where, in 2017, new HIV infections were highest in youths aged between 15 and 24 (1% annual incidence) with females having three times higher annual incidence than males (1.5% vs 0.5).^7^ Adolescent girls and young women are also at considerable risk of other negative sexual and reproductive health (SRH) outcomes including STIs and early pregnancy.^8^

Despite their increased risk of HIV infection, in South Africa, adolescent girls and young women in general, and those aged under 18 in particular, typically have low uptake of HIV and reproductive health services due to concerns around stigmatisation and limited provision of adolescent-friendly services.^2,9^ In rural KwaZulu-Natal, a setting in which adolescent girls’ and young women’s HIV incidence is high, prior to 2015 there existed few HIV prevention interventions specifically targeted at adolescents and youth.^2^ This may explain in part why despite high levels of awareness of where to receive antiretroviral treatment, HIV and reproductive health service uptake among adolescent girls and young women was low.^2^

It was into this context that in 2015, the DREAMS initiative was introduced. Funded by the US President’s Emergency Plan for AIDS Relief (PEPFAR) and implemented between April 2016 and September 2018, DREAMS is a multi-sectoral HIV prevention programme that envisions that young women should be Determined, Resilient, Empowered, AIDS-free, Mentored and Safe (DREAMS) and involves a combination of HIV prevention interventions that seek to target the multiple sources of risk that increase adolescent girls’ and young women’s vulnerability to HIV infection.^10,11^ DREAMS resulted in a considerable scaling-up of the SRH services available to young people in the implementing communities.

During the implementation of DREAMS between 2016 and 2018, an independent, evidence-based “edutainment” programme, *MTV Shuga Down South* Season V was broadcast on national television with one episode a week for 12 weeks (with repeats later the same week), over two consecutive years. It was also reinforced through MTV Shuga-related messaging through a “360 mass media” campaign with a strong presence on radio, social media, and comic books. The storylines, content, and experiences of characters on MTV-Shuga are co-produced with young people and based on formative research in each context in which the programme is set.^12^ Previous and subsequent seasons of MTV Shuga have been set in Kenya and Nigeria with plans to set future seasons in Egypt and India.

Based on Badura’s Social Learning Theory, which posits that people learn from interaction with others in social contexts,^13^ and Sabido’s theory of Tone in Human Communication, which establishes that it is possible to use entertainment to achieve a proven social benefit,^14^ edutainment programmes have been widely used throughout the world as a means to “educate” and “entertain” audiences and tackle serious social issues in bold and engaging ways. They are grounded in theories of narrative persuasion which illuminate the factors and processes that seek to facilitate changes in audience members’ knowledge, attitudes, and behaviours.^15^ The approach seeks to inform and influence individual (through sparking interest, holding attention, and reducing potential resistance to top-down advice) and social change (through shifting social norms).^16^ As part of edutainment, stories are designed to draw the audience into the centre of an issue and move them emotionally as a means of sparking reflection, discussion, and debate.^17^ The screening of MTV Shuga during the implementation of DREAMS, though independent of DREAMS, also provided an important opportunity to better ensure that services were available, particularly if the programme precipitated an increased demand for services amongst viewers.

While it can be argued that the educational element of edutainment interventions might be too diluted for viewers to recognise and retain the relevant information, or that their fictional nature may lead viewers to trust the quality of the information less than “official” sources, evaluations of edutainment programmes including *Intersexions* in South Africa, and *East Los High* in the United States have shown that such programmes can have wide audience reach, strong viewer engagement, and positive impacts on young people’s SRH.^15,16,18^ The MTV Shuga approach has also been shown to positively impact young viewers’ SRH outcomes.^16^ Through a randomised controlled trial conducted in urban Nigeria in 2013, young viewers were exposed to MTV Shuga or a placebo television series through study-organised screenings. The findings of this study suggest that eight months after the intervention, those who were exposed to MTV Shuga were twice as likely to test for HIV (as measured through the redemption of a voucher). Reductions in sexual transmitted infections (chlamydia), were also found among women who were exposed to MTV Shuga as was an improvement in treated individuals’ knowledge about topics covered during the series including sources of HIV transmission, awareness of antiretroviral therapy, and knowledge about the need to take a second test three months after the first (window period).^16^ The aim of this paper is to explore the ways in which MTV-Shuga Down South was reported to influence participants’ engagement with SRH information in a rural setting in KwaZulu-Natal, South Africa.

## Methods

### Study context

Through existing surveillance activities, a representative population-based prospective cohort of females aged 13–23 was established in 2017 and followed over two years in a rural and resource-constrained area, uMkhanyakude, a district in KwaZulu-Natal which is generally poorer than other parts of South Africa. In this paper, we report on a nested qualitative study that was conducted in the same population to explore the potential of mass-media edu-dramas to contribute to improving the sexual health of adolescent girls and young women. Community screenings of MTV-Shuga were held followed by explorative qualitative research.

This study was conducted in five communities, and although predominately rural, the area contains an urban township and informal, peri-urban settlements. Most households are multi-generational with an average size of 7.9 (SD4.7).^19^ Employment is low (over 85% youth (20–24) unemployed)^2^ and mobility is high, particularly among young people.^2,19^ HIV prevalence is high (41% antenatal HIV prevalence), and HIV incidence is 4.5% per annum in 15–19- year-old girls, and 7.5% per annum in 20–24-year-old women. Pregnancy rates are also high with 20.9% and 72.6% of 15–19 and 20–24-year-olds having ever been pregnant.^2^

### MTV Shuga Down South

In 2019 over a period of eight weeks, eight community-based screenings of a single 22 minute MTV-Shuga Down South episode were conducted. Each screening was independent of the others. Set in the cool clubs, hangouts, and schools of Johannesburg’s Braamfontein, and the township of “Zenzele”, MTV Shuga Down South was made up of 12 episodes. The season’s themes included abusive relationships, toxic masculinity, sexual assault, back alley abortions, death, and “blessers” (age-disparate transactional sexual relationships whereby older rich men (blessers) entice adolescent girls and young women (blessees) with money and gifts in exchange for sexual favours^20^) in addition to continuing the conversation about HIV.^21^ Any one of four episodes (episodes 4–7) were shown at the community screenings. The specific episodes that were screened were chosen as the content represented most of the thematic areas that were shown in the whole season.

### Sampling and data collection

Recruitment into the study was done in two stages. In the first stage, communities in which the screenings were conducted were sampled. Attempts were made to maximise the heterogeneity of these communities such that four screenings were conducted in schools and four in community settings in various geographical locations as described in Table 1. The locations for the screenings were selected to represent peri-urban, rural, and deep rural communities. In these communities, school and community venues were selected for viewings. Within schools, permission was sought from the school authority. Once permission was given, the research team was given an opportunity to inform learners about the screening of MTV SHUGA and about the dates of the viewing. Those who participated knew each other because they all came from the same school. In the community, permission for viewings was sought from church venues and community halls. Young people in those communities were then informed by the research team. Schools were also asked to inform young people about the community screenings. Young people residing closer to those venues usually came and this meant some were friends and knew each other. Informed consent was obtained from the parents or guardian of all participants aged below 18 years in advance of the screening and research. During the screenings, structured observations were conducted by trained social science research assistants (SSRAs) who noted any participant reactions to the content of the episodes which were then probed during the qualitative data collection activities that followed.

**Table 1.** Participants by age, sex, community, and research activity

Description of community	Activity				
	Screenings	IDI	FGD		
		f/m (ages)	Female (ages)	Male (ages)	Mixed female/male (ages)
Rural area with medium-sized population	1	5 f (15–16, 1 aged 28) 1 m (aged 19)			6f/2 m (15–19)
Peri-urban area with large population size	2	4 females (aged 15–16) 1 male (aged 16)	11 (14–22) 7 (15–19)		4f/8 m (16–19)
Rural area with mine and medium-sized population with high mobility	2	3 females (aged 15–23) 1 male (aged 21)	12 (18–22)	6 (13–23) 10 (18–26)	
Rural area with large population	2	4 females (aged 16–21) 1 male (aged 28)	9 (16–30) 9 (15–19)	10 (18–23) 9 (16–18)	
Deep rural area	1	4 females (aged 15–20) 1 male (aged 15)	12 (17–20)	11 (18–23)	
Total	8	25	13		

In the second stage, and as summarised in Table 1, following the screenings, 25 in-depth interviews (IDIs) and 13 focus group discussions (FGDs) with a total of 126 participants were conducted with female and male participants. IDI participants ranged in age from 15 to 28 and FGD participants ranged in age between 15 and 30 years. Sampling for the research activities was purposive. Inclusion criteria were that participants fell into the target age range, were residents of a sampled community or students in a sampled school, and that they were able to give informed consent (for those 18 and over) or assent (for those under 18) to attend the screening and participate in the study. Individuals who had previously watched, or otherwise engaged with, MTV Shuga were not disqualified from attending a screening or participating in the research activities. Communities and schools were purposively sampled to maximise the heterogeneity and geographical spread of the sample. A qualitative description of the communities is included in Table 1. All interviews were conducted in isiZulu using a semi-structured topic guide and audio recorded.

Interviews were conducted by four trained SSRAs who had extensive experience on research in the study context, on interviewing on sensitive topics and with vulnerable populations. SSRAs underwent specific training on the study protocol, tools, and ethical procedures. Topic guides were developed based on the objectives of the study and review of the literature. Themes included young people’s lives in their communities, their perceptions and understandings of sexual health and HIV, their social and peer networks, their viewing habits and their perceptions, views and experiences of MTV-Shuga. All topic guides were piloted after which no significant changes were made.

### Analysis

Full-team debriefing sessions were held at regular intervals to discuss the data, reflect on emerging themes, explore potential new lines of enquiry, and evaluate any unexpected findings. Completed interviews were transcribed verbatim and translated into English for analysis. Data analysis was thematic and complemented by constant comparison and deviant case analysis techniques.^22^ Based on the topic guides, the review of the literature and any themes that emerged during the debriefs, the first author developed separate coding frames for the IDI and FGD interviews. These coding frames were used to reduce the data and summarise it by theme with coding being conducted by SSRAs who had collected the data. Coded data were read intensively and analysed by theme, comparing findings across setting, sex, and data generation method. No systematic differences in participants’ narratives were noted by data generation method. Below we summarise the model of the data that emerged from the analysis process. Any names used are pseudonyms.

### Ethical considerations

Participants aged 18 years or older provided written informed consent. Participants aged 14–17 years provided written informed assent after written informed consent for their participation was obtained from a parent or guardian. Ethical approval was provided by the ethics committees of the University of KwaZulu-Natal (reference BFC 339/16, 11 October 2018), and the London School of Hygiene and Tropical Medicine (reference 16886, 25 April 2019). All study participants were eligible for referral if either they requested one or a member of research or study staff thought that they would benefit from one. Referral services were provided by DREAMS implementing partners and included psychosocial support and counselling, post-exposure prophylaxis, emergency contraception, voluntary counselling and testing, and intimate partner violence support services. No participants were identified to require a referral.

## Results

In the results that follow we first briefly outline participants’ descriptions of their lives in their communities including their access to SRH information. We then examine participants’ narratives concerning what they learnt from MTV with relation to SRH.

### Young people’s lives and access to SRH information and services

Participants’ narratives of their lives and communities varied. While some described a relatively carefree life, mainly revolving around school and socialising, others’ narratives focussed more on the challenges that young people faced in their communities:

“*I have observed that young people of this community are alcoholics. There is drug usage and marijuana because they even smoke it here at school. Teenage pregnancy rate is also too high. There are pregnant teens left and right and some of them drop out of school. There is a lot to say but drug abuse is at the core.*” (FGD in peri-urban area with females aged 14–22)

This reflected a perception that some young people lived relatively chaotic lives which were also characterised by a lack of opportunities owing to both personal and structural barriers:

“*Some do not have money to further their studies after matric (final year of high school) because their families cannot afford to send them to university. Also, young people are not entrusted with higher positions as compared to Whites and Indians.*” (FGD in peri-urban area with females aged 14–22)

A few participants linked a lack of opportunities to SRH risks, particularly for girls:

“*Girls who are using drugs escape from home at night and go for sex work, they make a lot of money out of that and they use it to buy drugs. So, parents and sex work are sources of this money.*” (FGD in peri-urban area with females aged 14–22)

These challenges notwithstanding, many participants, both female and male, described having access to SRH information and services including from local clinics, social workers, as part of a study subject in school, and from the Africa Health Research Institute (AHRI):

“*I have information on what you should do to protect yourself when having sexual intercourse, you should condomise to prevent pregnancy and diseases … and abstain so that you won’t get HIV. From the clinic, AHRI also visits people’s household to help and educate people about things they don’t know. We also get it from Life Orientation subject at school, when teachers educate about the chapter that talks of health and sexual health.*” (IDI in rural area with mine and high population mobility with single female aged 19)

Some participants also described knowing how and where to access specific services such as contraception, HIV testing, and voluntary medical male circumcision (VMMC). Indeed, one male participant also accurately reflected on the fact that VMMC did not obviate the need for condoms given the risk of HIV infection even for those who are circumcised. Knowledge such as this suggests that while some participants also described receiving information from peers, in general, young people knew where they could access information concerning SRH risks and services in their local area. In practice however, few participants described feeling comfortable and confident to access this SRH information or services. Girls in particular described the challenges they experienced in accessing services owing to perceived judgement and disapproval by clinic staff of them being sexually active at their age (typically below 18).

“*No no no (shaking her head) not at all! It is not user friendly because if a teenager visits the clinic to test for pregnancy, nurses shout at you. Nurses shouted at another girl who went to the clinic to test for pregnancy, they judged her without even considering that maybe she is in that situation because she was raped. Maybe they can access them at that clinic but not [name of clinic] (both laugh), not here because nurses of [name of clinic] are so judgemental.*” (IDI in rural area with single female aged 15)

Furthermore, few participants described being able to approach their parents or guardians for SRH information or guidance even though several expressed a desire for this from their parents:

“*They normally talk with their peers, because you can’t just talk about this thing (sex) with your mother. It’s because you can’t just talk with your parent about this, he/she will beat you saying you don’t take him/her seriously … you can’t just go by yourself and ask your parent about sex, but it is a responsibility of a parent to call his/her child and explain to him/her about the consequences of having sex if you are still young. But our parents don’t exercise that responsibility, maybe they think if they talk with us about it, we would like to experience it whereas it is essential that we get enough information and facts about it because these things do happen.*” (IDI in peri-urban area with single female aged 15)

### MTV-Shuga and young people’s access to SRH information

#### Access to MTV-Shuga

In general, the majority of participants had not watched MTV-Shuga when it was shown on TV for reasons including preferring to watch other programmes; competition for the TV by programmes watched by other family members; the late hour at which it was screened on terrestrial television (even though it was moved to the earlier hour of 8 pm when the episodes were repeated); and not having DSTV Cable TV, access to data or Wi-Fi to watch it on YouTube, or a suitable device on which to watch it at a time of their choice. Those who had watched it were more often residents of the peri-urban community and from seemingly better-off households to the extent that they were able to afford the costs associated with DSTV Cable TV subscriptions and Wi-Fi at home.

Another reason that a number of participants – both female and male – gave for not having watched MTV-Shuga, despite being interested in it, was discomfort with watching it in the presence of the adults in their households and not having space or time to watch it by themselves:

“*I have watched MTV Shuga but grandmother immediately entered at the door, I decided to switch it off because it concerned condoms and I wasn’t comfortable when grandmother entered. It is not something comfortable to watch, because I like my space. I would prefer watching it alone, it is something great to watch sometimes. And I cannot download it because my mother will see it on my phone.*” (IDI in deep rural area with single male aged 15)

This also reflects the reluctance that some, particularly younger, participants expressed with discussing sex with the adults in their lives given that they feared that talking to them about sex could be perceived by their parents as a sign of disrespect and thus attract chastisement or punishment, as elaborated above.

#### The value and salience of MTV-Shuga

Following the screening, which was the first time that the majority of participants had watched MTV Shuga, a number of them described valuing MTV-Shuga because it was both educational – giving them an opportunity to engage with SRH information in a new way – and targeted specifically at young people. By watching the experiences and consequences of the decisions that the young characters in the episode made, study participants described learning something that they could relate to in their lives. To illustrate for example, following the experience of one of the main characters who started a sexual relationship before they were ready to, a number of younger participants described reflecting on the value of delayed sexual debut for those who were both in-and-out-of-school:

“*I learnt that if you are still at school or even if you are not at school, you should refrain from getting involved in sexual intercourse, you shouldn’t rush it because you will reach it, it won’t go anywhere or end.*” (FGD in rural area with large population with males aged 16–18)

Being able to see how their desire to simply “have fun” with their peers could affect their lives and futures was also described by some to be compelling, and a useful way of helping adolescent girls resist the pressure from peers to take risks:

“*I think it [MTV-Shuga] would bring change. The way girls are acting shows that there is peer pressure, like telling each other to go to night clubs … I think [the episode] shows a good example on what will happen at the end if you don’t listen. [You could] be just like Nomzamo [a character], who ended up having a baby just because she did not listen to her parents. Any girl would realize that she is doing wrong, and things will end up badly.*” (FGD in peri-urban area with females aged 14–22)

Similarly, a number of girls also reflected on the risk associated with intergenerational sex. Generally, these risks were described to include the risk of contracting HIV, the difficulty they faced in attempting to negotiate condom use with older male partners, and non-use of health services due to fear of their community or health care workers knowing that they were involved with an older person:

“*Yes, I got [from MTV-Shuga] that as a school student, you mustn’t have feelings for older people and shouldn’t be a student that goes after the teachers … when you start engaging in sexual intercourse, you should know, what you are doing and at what time, you should know, what will happen if you fall pregnant or maybe what will help you if you get infected with HIV.*” (FGD in rural area with large population with females aged 16–18)

Previous research in the setting that has revealed that intergenerational sex is a stigmatised behaviour would also likely explain why no participants described being involved with an older man even though they agreed that within their community they had seen “other” young girls being involved in such relationships.^23^

It is in the context of this stigmatised behaviour, and reflecting the experiences of one of the characters featured in the episode, that some female participants described MTV-Shuga as helping adolescent girls to avoid transactional sex as a way to meet their needs.

“*I think MTV Shuga has a good effect because it educates us not to offer ourselves to anyone, and that we shouldn’t be attracted to someone who has money, to do things we want for us.*” (FGD in rural area with large population with females aged 16–18)

Having watched an episode, a number of younger participants, both female and male, also reflected on the role that alcohol could play in exposing them to harm by compromising their ability to give consent and through making them vulnerable to the ill intentions of others:

“*I saw on that episode that they were doing wrong things … [one of the characters] was getting his girlfriend drunk because he wanted to have sex with her without her consent, that’s why he was serving her with alcohol because she didn’t agree.*” (FGD in rural area with large population with females aged 17–20)

While many participants described having access to SRH information, including about the risks of unprotected sex, the negative role and consequences of drinking alcohol or having sex while drunk (as evidenced in the episode), a storyline around alcohol appeared to hold particular salience for participants. This may have been because of the sobering effect of seeing how misusing alcohol could negatively affect their lives, particularly considering alcohol’s association with fun and socialising.

Adolescent boys and young men also described the value they derived from having watched MTV-Shuga:

“*What I saw from the story is that if you have your own women (a girlfriend) you must be protected and do protected sex by using a condom.*” (FGD in deep rural area with large population with males aged 16–18)

Seeing how not using a condom could negatively affect their lives may have been particularly salient for adolescent boys and young men, given the invincibility of youth and masculinity norms that rewarded them for having girlfriends and sexual conquests. A few also reflected on how the behaviour of boys could negatively affect girls, suggesting that watching the experiences of female character increased their awareness and empathy:

“*If they are aware that a girl behaves irresponsibly, they take advantage of that and have sex with her and then leave her. They will then tell their friends who will come back to do the same thing.*” (FGD in rural area with large population with males aged 16–18)

The featured storylines appeared to be salient for participants owing to them being considered believable. This relatability was described to come from the fact that they were able to recognise the stories and draw parallels in their lives or the experiences of others that they knew in their communities. In this way, watching the experiences of individual characters provided them with a guide to see how their decisions could similarly affect them or others like them:

“*Yes, they [storylines] are believable … there is somebody I know … who did something like that … she was drinking [a lot]  … with her friends … and then her boyfriend had sex with her and took a video, that video was all over social media and was seen by the whole school, this female was hurt by this because it caused her to quit school.*” (IDI in deep rural area with single female aged 15)

For many participants, the value of MTV-Shuga lay in the fact that it addressed issues of concern to them, issues that their parents did not discuss with them. Indeed, many participants, particularly female participants, shared that they would recommend MTV-Shuga to others:

“*It was a great thing that they showed it to us. And if it was up to me, MTV Shuga should have been shown to all young people because it shows the things they would experience in life as young females and as a youth in general. I liked everything about it, and I wish that every person may watch. I will also tell/ recommend other people to watch it and I will start with my younger sister, although she is still young but I will ask her to watch it.*” (IDI in peri-urban area with single female aged 15)

The value of MTV-Shuga also seemed to be derived in its portrayal of the challenges that young people face, which were addressed at the inter-personal, relationship level, rather than as a subject at school or information that they would receive from a clinic. Furthermore, by being able to see how their decisions may affect their lives, MTV-Shuga provided valuable learnings from the experiences of characters in the episode:

“*You learn something from everything you watch, you could even learn something from Nomzamo’s bad behaviour. When Nomzamo does wrong things, you can learn that doing wrong things may have a negative impact on you.*” (FGD in peri-urban area with large population with females aged 14–22)

MTV-Shuga was also valued for raising participants’ awareness of what to do in the face of challenging and difficult situations especially through modelling such actions:

“*It [MTV-Shuga] educates that if you’re a person maybe a girl and find yourself raped you must report the case and not to keep silent about it.*” (FGD in deep rural area with females aged 17–20)

While participants may have been aware of these options, watching a character actually grappling with these issues and actually seeking help may have acted to demystify what was involved in reporting, and perhaps raise individuals’ comfort with doing so were they to find themselves in a similar situation.

## Discussion

This paper provides evidence that MTV-Shuga was well-received and resonated with participants in a low-income setting in KwaZulu-Natal. By exploring what participants say they learnt, this article focuses on the most salient aspects of the MTV-Shuga episode that they viewed, and what this means for adolescents’ and young people’s SRH. Discussion of the pathways of effect – or *how –* MTV-Shuga was described to impact on young people’s behaviour as they navigate HIV prevention and SRH services, is the subject of a forthcoming publication from this study.

The findings of this study highlight that in this rural, poor, high HIV-burden setting in which young people face risks and vulnerabilities arising from personal and structural barriers,^8^ young people exhibited high levels of awareness of information on SRH and HIV. Uptake of available SRH services, however, has historically been low.^2^ This was described, in part, to be as a result of unmarried adolescent girls’ perceived experience of judgement and disapproval by clinic staff for being sexually active, and a lack of adolescent-friendly services.^2^ MTV Shuga presented scenarios to young people which offered possibilities for acting on their knowledge in their own real-life situations. These included ways of resisting peer pressure to have fun and take potentially harmful risks; strategies for rejecting harmful social norms that persuade adolescent girls and young women to engage in transactional sex as a means to meet their needs;^24,25^ and making their own decisions having thought through the potential consequences of their choices for their future. In this way, MTV Shuga provided an entertaining and visual guide for young people to navigate the risk and vulnerabilities that they faced in a way that engaged and resonated with them. While the programme was set in the urban setting of Johannesburg and a surrounding township, this did not seem to affect the relatability of the issues discussed by participants given that the SRH risks and vulnerabilities that adolescents face in rural KwaZulu-Natal are similar. The extent to which young people would actually act with the knowledge they gained after having had the opportunity to watch only one episode, however, is unclear.

Unlike other edutainment interventions that describe wide coverage and strong audience engagement,^18,15^ our findings illustrate that owing to a variety of practical reasons, the majority of participants had not watched MTV-Shuga when it was broadcast on public television (and repeated the same week) or on YouTube. Many participants also described feeling uncomfortable or unable to watch the programme with older relatives, and also lacking time, space, or resources to watch it on their own. This implies that participants desired space away from adults to watch the programme, with some watching it in secret. Some participants also expressed interest in telling their peers about MTV Shuga or watching it with them. This implies a clear potential to provide more opportunities for young people to watch and discuss MTV Shuga with their peers outside of their homes in safe spaces such as youth clubs, at school, or through community or NGO-hosted screenings. With a clear interest in watching MTV-Shuga, and a stated desire by some participants for wanting to receive guidance and information from adults in their lives, these findings also suggest that there would likely be an opportunity for MTV-Shuga, as part of its mode of delivery, to act as a conduit for young people to discuss sex with adults, by encouraging parents to watch it as a way to open up communication about sex with their children. This could be by making parents aware of the ambition and potential of MTV and providing guidance on having structured conversations within families about SRH. This has been described as a positive outcome of other edutainment programmes including Intersexions which was found to create space for a new level of openness in a number of family relationships enabling more honest and meaningful discussions about SRH issues with children.^18^

Our findings also describe the value that participants gave to MTV-Shuga’s approach of being both educational and specifically targeted at young people. Participants described learning about how to navigate, manage and respond to risks associated with early sexual debut, inconsistent condom use, alcohol misuse, and intergenerational transactional sex relationships. Given that participants would likely have been introduced to some of these risks through various channels including through school, the Africa Health Research Institute, local clinics, and social workers, our findings suggest that accessing the information in this new and engaging way made the information more salient. Similarly, being able to watch the experiences of individual characters provided them with a guide to see how their own decisions could affect them. By situating these risks within “relationships” rather than academic or medicalised didactic information, and through being able to watch the experiences of young people like them and the consequences of the decisions they made, participants also described the programme as being believable and relatable to them and their lives. This finding has also been noted through the evaluation of other edutainment programmes such as *Intersexions* in South Africa.^18^

### Limitations

This study has strengths and limitations. The wide range of geographical locations from which individuals were sampled contributed to maximising the heterogeneity of the sample. This was particularly important for ensuring that young people who had previously been unable to watch MTV-Shuga were included. A few of the FGDs were however quite large in size (up to 12 participants) or had a broad age range and two were of mixed sex. This may have reduced the willingness of some participants to speak candidly about their views. The effect of this was minimised through the extensive experience of the SSRAs who conducted the interviews and strict adherence to ethical protocols to ensure that participants only answered questions that were of their comfort level. The fact that the public screenings were limited to one episode per area also meant that some story arcs were not resolved for participants. This meant that these storylines and the potential consequences of characters’ decisions could not be fully explored, potentially resulting in gaps in participants’ understanding, and thus ability, to engage with the themes and reflect on how their own decisions could potentially affect their own lives. We also acknowledge that the structure of the data collection – such that the FGD and IDIs were conducted after the viewing – may have enabled participants to engage and reflect with the content of the episode more deeply than had they just watched it simply as a programme in their everyday lives and in the context of everyday distractions. On the other hand, it also highlighted the value of promoting dialogue around the storylines and themes and creating opportunities for individuals to watch the programme with others rather than alone.

## Conclusion

MTV-Shuga appears to have had particular salience amongst adolescents, enabling them to animate important and accurate information related to their SRH owing to its educational and youth-friendly design and targeting. There is a valuable potential to make MTV-Shuga more accessible to young people in rural areas with limited access to TV and internet through safe spaces, as well as to make parents more aware of MTV Shuga. If augmented by structured discussions following the viewings (with peers and parents), MTV Shuga may act as a valuable educational resource helping individuals and communities to challenge negative social norms and improve discussion about SRH for the benefit of young people.

## References

[1] UNICEF. (2020). HIV and AIDS in adolescents. Retrieved 17/02/2021. Available from: https://data.unicef.org/topic/adolescents/hiv-aids/.

[2] Chimbindi N, Mthiyane N, Birdthistle I, et al. Persistently high incidence of HIV and poor service uptake in adolescent girls and young women in rural KwaZulu-Natal, South Africa prior to DREAMS. PLoS One. 2018;13(10):e0203193. 30325932 10.1371/journal.pone.0203193 PMC6191091

[3] UNAIDS. Adolescents, young people and HIV. The GAP Report 2014. Geneva: UNAIDS; 2014.

[4] Dellar RC, Dlamini S, Abdool Karim Q. (2015). Adolescent girls and young women: key populations for HIV epidemic control. 10.7448/IAS.18.2.19408 PMC4344544 25724504

[5] Fielding-Miller R, Dunkle KL, Jama-Shai N, et al. The feminine ideal and transactional sex: navigating respectability and risk in Swaziland. Soc Sci Med. 2016;158:24–33. 27107148 10.1016/j.socscimed.2016.04.005 PMC4875790

[6] UNAIDS (2010). Adolescents, young people and HIV. UNAIDS Fact Sheet. Geneva: UNAIDS.

[7] Musekiwa A, Bamogo A, Shisana O, et al. Prevalence of self-reported HIV testing and associated factors among adolescent girls and young women in South Africa: results from a 2017 nationally representative population-based HIV survey. Public Health in Practice. 2021;2:100093. 36101581 10.1016/j.puhip.2021.100093 PMC9461291

[8] Zuma T, Seeley J, Mdluli S, et al. Young people’s experiences of sexual and reproductive health interventions in rural KwaZulu-Natal, South Africa. Int J Adolesc Youth. 2020;25(1):1058–1075. 34177039 10.1080/02673843.2020.1831558 PMC8224946

[9] Ayton SG, Pavlicova M, Abdool Karim Q. Identification of adolescent girls and young women for targeted HIV prevention: a new risk scoring tool in KwaZulu Natal, South Africa. Sci Rep. 2020;10(1):13017. 32747693 10.1038/s41598-020-69842-x PMC7400571

[10] Chimbindi N, Birdthistle I, Shahmanesh M, et al. Translating DREAMS into practice: early lessons from implementation in six settings. PLOS ONE. 2018;13(12):e0208243. 30543640 10.1371/journal.pone.0208243 PMC6292585

[11] Saul J, Bachman G, Allen S, et al. The DREAMS core package of interventions: a comprehensive approach to preventing HIV among adolescent girls and young women. PLOS ONE. 2018;13(12):e0208167. 30532210 10.1371/journal.pone.0208167 PMC6285267

[12] Levy J. (2015). MTV Shuga – a multi-platform communication initiative achieving HIV behaviour change for adolescents in Africa. Retrieved 06/12/2021. Available from: https://www.comminit.com/la/content/mtv-shuga-multi-platform-communication-initiative-achieving-hiv-behaviour-change-adolesc.

[13] Bandura A. Self-efficacy: toward a unifying theory of behavioral change. Advances in Behaviour Research and Therapy. 1978;1(4):139–161.

[14] Sabido M. Miguel Sabido’s entertainment-education. In: LB Frank, P Falzone, editors. Entertainment-education behind the scenes: case studies for theory and practice. Cham: Springer International Publishing; 2021. p. 15–21.

[15] Wang H, Singhal A. East Los High: transmedia edutainment to promote the sexual and reproductive health of young Latina/o Americans. Am J Public Health. 2016;106(6):1002–1010. 27077336 10.2105/AJPH.2016.303072 PMC4880269

[16] Banerjee A, Ferrara EL, Orozco-Olvera VH. The entertaining way to behavioral changefighting HIV with MTV. Policy research working paper. Development impact evaluation group. Washington (DC): World Bank; 2019. p. 8998

[17] Perlman H. Edutainmnet: using stories and media for social action and behavioural change. Johannesburg: Soul City Institute; 2013.

[18] Myers L, Hajiyiannis H, Clarfelt A, et al. Evaluation of season two of the television drama series, intersexions. Johns Hopkins Health and Education in South Africa (JHHESA); 2014.

[19] Tanser F, Hosegood V, Bärnighausen T, et al. Cohort profile: Africa centre demographic information system (ACDIS) and population-based HIV survey. Int J Epidemiol. 2008;37(5):956–962. 17998242 10.1093/ije/dym211 PMC2557060

[20] Mampane JN. Exploring the “blesser and blessee” phenomenon: young women, transactional sex, and HIV in rural South Africa. SAGE Open. 2018;8(4):1–9.

[21] MTV Shuga. (2018). MTV Shuga Down South Season V. Retrieved 17/02/2021. Available from: https://www.mtvshuga.com/show/?series = series-5.

[22] Glaser BG, Strauss AL. The discovery of grounded theory: strategies for qualitative research. Chigaco (IL): Aldine; 1967.

[23] Kyegombe N, Stoebenau K, Chimbindi N, et al. Measuring transactional sex in different contexts: how do tools to measure this practice perform in rural South Africa? Afr J AIDS Res. 2021;20(4):329–335. In Press. 34905457 10.2989/16085906.2021.2012213

[24] Mojola SA. Love, money and HIV: becoming a modern African woman in the age of AIDS. California: University of California Press; 2014.

[25] Kyegombe N, Meiksin R, Wamoyi J, et al. Sexual health of adolescent girls and young women in Central Uganda: exploring perceived coercive aspects of transactional sex. Sex Reprod Health Matters. 2020;28(1):1700770. 31934824 10.1080/26410397.2019.1700770 PMC7888006

